# Draft Genome Sequence of the Beer Spoilage Bacterium *Megasphaera cerevisiae* Strain PAT 1^T^

**DOI:** 10.1128/genomeA.01045-15

**Published:** 2015-09-10

**Authors:** Kirthi K. Kutumbaka, Joshua Pasmowitz, James Mategko, Dindo Reyes, Alex Friedrich, Sukkyun Han, Willm Martens-Habbena, Jason Neal-McKinney, Harish K. Janagama, Cesar Nadala, Mansour Samadpour

**Affiliations:** Institute for Environmental Health (IEH), Lake Forest Park, Washington, USA

## Abstract

The genus *Megasphaera* harbors important spoilage organisms that cause beer spoilage by producing off flavors, undesirable aroma, and turbidity. *Megasphaera cerevisiae* is mainly found in nonpasteurized low-alcohol beer. In this study, we report the draft genome of the type strain of the genus, *M. cerevisiae* strain PAT 1^T^.

## GENOME ANNOUNCEMENT

Improvements in modern brewing technology have resulted in beer with significantly reduced oxygen content, allowing growth of strictly anaerobic microorganisms such as *Megasphaera* sp. and *Pectinatus* sp. (1, 2). *M. cerevisiae* alone was found responsible for 3 to 7% of beer spoilage cases in Europe between 1980 and 2002 (3, 4). Contamination of unpasteurized beer by *M. cerevisiae* and *Pectinatus* sp. causes high turbidity and off taste due to a considerable accumulation of butyric acid along with smaller amounts of acetic, isovaleric, valeric, and caproic acid, as well as acetoin (5, 6). The type strain of the genus, *M. cerevisiae* strain PAT 1^T^, was isolated from spoiled bottled beer in 1985 (7).

*M. cerevisiae* strain PAT 1^T^ (DSM 20462 = ATCC-43254) was obtained from the German Collection of Microorganisms and Cell Cultures (DSMZ, Braunschweig, Germany). The strain was grown in modified PYG medium (DSMZ) anaerobically at 30°C until early stationary phase. Genomic DNA was isolated for whole-genome sequencing using the bacterial DNA purification kit (Pi Biologique, Seattle, WA, USA). The genome library was then prepared using the Nextera XT DNA sample prep kit (Illumina, San Diego, CA, USA). Genome sequencing was performed using an Illumina MiSeq desktop sequencer (Illumina) with a paired-end 2 × 250 cycle MiSeq reagent kit. The 1,548,102 generated shotgun reads were assembled using the A5-miseq assembly software (8), resulting in 203 contigs with 90-fold coverage. The draft genome was annotated using Rapid Annotations using Subsystems Technology version 2.0 (RAST) (9, 10) and the NCBI Prokaryotic Genome Annotation Pipeline (PGAP) (11). The draft sequence has a total length of 3,238,021 bp, an average GC content of 44.8%, an *N*_50_ length of 49,680 bp, and a maximum contig size of 154,499 bp. Based on PGAP, the genome contains 17 rRNAs, 54 tRNAs, and 3,017 protein-coding genes. Using the PHAge search tool (PHAST) (12), we found seven prophage regions, of which two were intact (23.2 kb and 55 kb) and five were incomplete (28.1 kb, 11.9 kb, 38.7 kb, 20.2 kb, and 9.8 kb). RAST annotation identified CRISPRs and its associated elements on multiple scaffolds.

In well-studied beer spoilage bacteria, such as *Lactobacillus brevis,* catabolism of arginine and resistance to antimicrobial compounds from hops have been identified as key factors contributing to growth in beer and wine (13, 14). Arginine is catabolized using the arginine deiminase pathway through a series of metabolic reactions performed by arginine deiminase (*arcA*), ornithine transcarbamoylase (*arcB*), and carbamate kinase (*arcC*), leading to putrescine accumulation. The *M. cerevisiae* draft genome encodes an intact *arcABC* operon, suggesting that it can catabolize arginine. Hop compounds found in beer exhibit antimicrobial effects because of their ability to lower the pH and sequester divalent metal cations such as iron and manganese (15). The *M. cerevisiae* draft genome encodes several operons implicated in iron homeostasis, such as the *fur* and *suf* operons, as well as an ABC-type transporter (*mntH*) that is critical for manganese uptake. Taken together, these observations support that *M. cerevisiae* is well equipped to replicate and grow in beer and cause its spoilage.

### Nucleotide sequence accession number. 

The *M. cerevisiae* strain PAT1^T^ genome sequence was deposited in GenBank under the accession number LEKT00000000.
